# Association of MASLD with chronic kidney disease according to metabolic profiles and fibrosis severity: a KNHANES analysis

**DOI:** 10.3389/fendo.2026.1923883

**Published:** 2026-09-01

**Authors:** Hye Jin Park, Soo Wan Kim

**Affiliations:** 1 Department of Internal Medicine, Chonnam National University Hospital, Gwangju, Republic of Korea; 2 Department of Internal Medicine, Chonnam National University Medical School, Gwangju, Republic of Korea

**Keywords:** cardiometabolic risk factors, chronic kidney disease, diabetes mellitus, hypertension, liver fibrosis, metabolic dysfunction-associated steatotic liver disease

## Abstract

**Background:**

Metabolic dysfunction-associated steatotic liver disease (MASLD) is increasingly recognized as a multisystem metabolic disorder with extrahepatic manifestations, including chronic kidney disease (CKD). However, population-level evidence on the renal impact of metabolic heterogeneity and fibrosis severity in MASLD remains limited. We aimed to evaluate the association between MASLD and prevalent CKD and to examine the contributions of metabolic components and liver fibrosis severity.

**Methods:**

We analyzed 17,229 adults (≥19 years) from the Korea National Health and Nutrition Examination Survey (KNHANES) 2011–2014. MASLD was defined as hepatic steatosis (hepatic steatosis index ≥36) with at least one cardiometabolic risk factor. CKD was defined as estimated glomerular filtration rate <60 mL/min/1.73 m² or urine albumin-to-creatinine ratio ≥30 mg/g. Survey-weighted logistic regression models estimated odds ratios (ORs) for CKD. Additive interaction between hypertension and diabetes was quantified using relative excess risk due to interaction (RERI) and attributable proportion (AP). Liver fibrosis severity was assessed using the log-transformed Fibrosis-4 index and restricted cubic spline analyses.

**Results:**

MASLD was associated with CKD (adjusted OR, 2.47; 95% CI, 1.76–3.47; p<0.001). Within MASLD, hypertension and diabetes were consistently associated with CKD. While point estimates suggested a positive additive interaction between these factors (RERI = 0.60; AP = 0.10), it did not reach statistical significance. Increased fibrosis severity showed a graded association with CKD, particularly among middle-aged adults.

**Conclusion:**

MASLD was associated with prevalent CKD. The association with prevalent CKD within MASLD varied according to metabolic profiles and fibrosis severity, highlighting the importance of risk stratification and integrated liver–kidney management.

## 1 Introduction

In 2023, a multinational Delphi consensus introduced the term metabolic dysfunction–associated steatotic liver disease (MASLD) to replace non-alcoholic fatty liver disease (NAFLD) (1). This transition represents a fundamental shift in the clinical paradigm; unlike the previous NAFLD definition, which relied on the exclusion of significant alcohol consumption, the MASLD criteria emphasize the presence of cardiometabolic risk factors as key determinants of hepatic steatosis (2). Currently, MASLD is the most common chronic liver disease, affecting over 30% of adults worldwide, with its burden steadily increasing alongside rising trends in obesity, type 2 diabetes, and metabolic syndrome (3, 4). Consequently, MASLD is now recognized not merely as a localized hepatic condition, but as a multisystemic metabolic disorder with profound extrahepatic implications (5).

Among the extrahepatic conditions associated with MASLD, chronic kidney disease (CKD) remains a critical global health concern due to its strong association with cardiovascular morbidity and mortality (6, 7). While numerous observational studies and meta-analyses have established a link between the former definition of NAFLD and incident CKD (8, 9), emerging data applying the new MASLD criteria suggest that the cumulative burden of metabolic dysfunction and liver fibrosis further escalates this risk (10–12).

Despite these advancements, a significant gap remains in the current literature. Much of the existing evidence is derived from hospital-based cohorts or specific health-screening populations, which may be subject to selection bias and may not accurately reflect the broader general population. Furthermore, the prevalence of CKD across the heterogeneous spectrum of MASLD, particularly according to metabolic components and fibrosis severity, remains insufficiently characterized in community-dwelling populations.

To address this limitation, we conducted a population-based analysis using nationally representative data from the Korea National Health and Nutrition Examination Survey (KNHANES). The KNHANES database offers a distinct methodological advantage by incorporating a complex, stratified, multistage probability sampling design of community-dwelling adults. This approach enables a robust estimation of disease burden at the national level while significantly minimizing the selection bias inherent in hospital-based studies (13). In this study, we investigated the association between MASLD and prevalent CKD in a nationally representative Korean population. We further examined the contributions of individual metabolic dysfunction components and liver fibrosis severity to CKD risk within the MASLD spectrum.

## 2 Patients and methods

### 2.1 Data source and study population

The data for this study were derived from the Korea National Health and Nutrition Examination Survey (KNHANES) V (2011–2012) and VI (2013–2014). KNHANES is a nationwide, population-based, cross-sectional survey conducted by the Korea Disease Control and Prevention Agency (KDCA) to monitor the health status, health-related behaviors, and nutritional conditions of the non-institutionalized Korean population. The survey employs a stratified, multistage probability sampling design to ensure national representativeness and includes standardized health interviews, health examinations, and laboratory assessments.

This study utilized the Korea National Health and Nutrition Examination Survey (KNHANES) data, which are publicly available and anonymized. The study was exempted from full Institutional Review Board (IRB) review by the Institutional Review Board of Chonnam National University Hospital (IRB No. CNUH-EXP-2026-065), as it involved the analysis of pre-existing, de-identified data. This study was conducted in accordance with the Declaration of Helsinki and reported following the STROBE guidelines.

**Figure 1:**
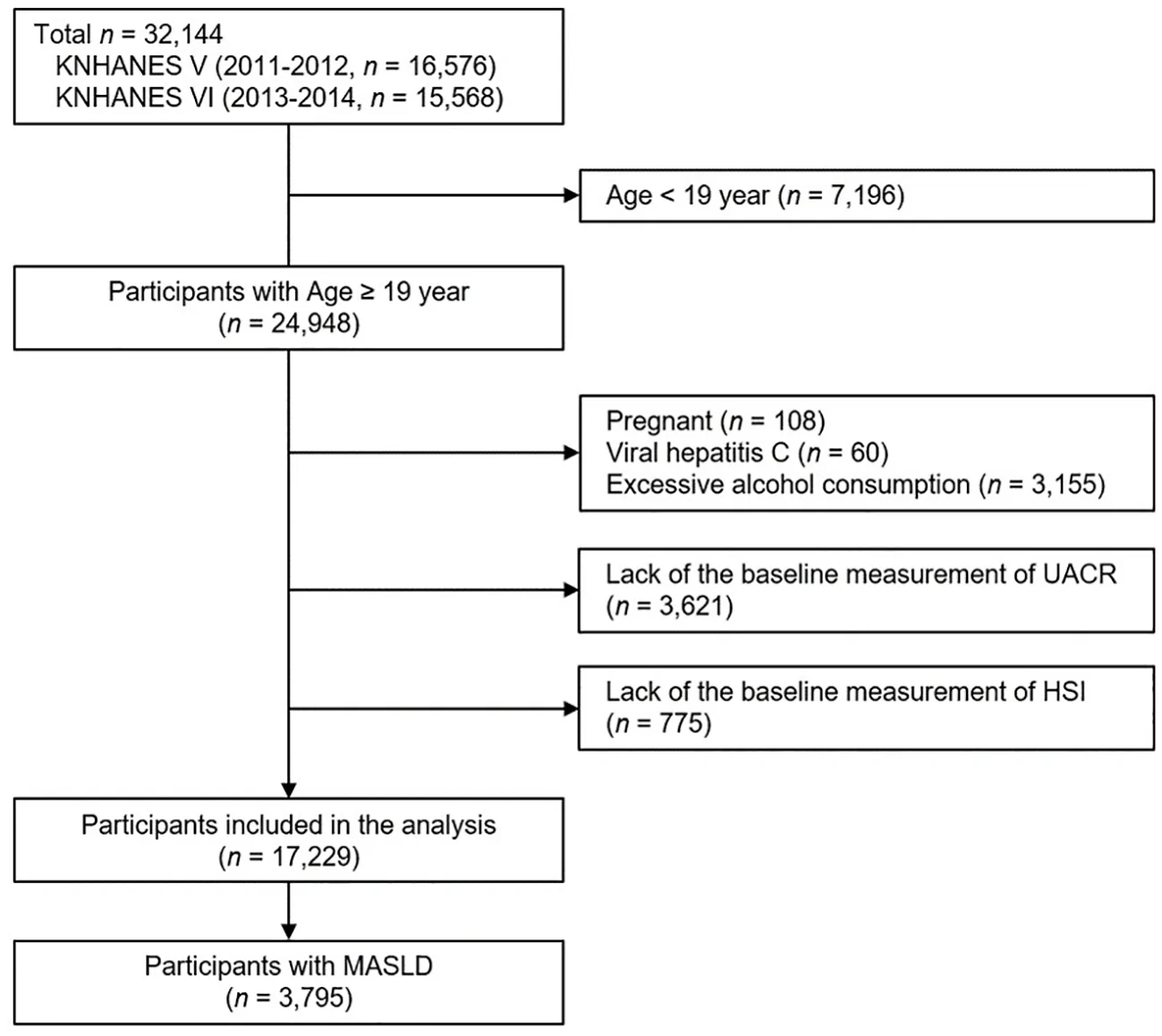
Flowchart of the study population selection. The flowchart shows the inclusion and exclusion process of participants from the Korea National Health and Nutrition Examination Survey (KNHANES) 2011–2014. KNHANES, Korea National Health and Nutrition Examination Survey; UACR, urine albumin-to-creatinine ratio; HSI, hepatic steatosis index; MASLD, metabolic dysfunction-associated steatotic liver disease.

From an initial pool of 32,144 participants, we identified 24,948 adults aged ≥19 years. Individuals were excluded if they met the following criteria: pregnancy (n = 108), positive status for viral hepatitis C (n = 60), or excessive alcohol consumption (n = 3,155), defined as an average daily alcohol intake of ≥30 g for men and ≥20 g for women. Furthermore, we excluded those missing baseline data for the urine albumin-to-creatinine ratio (UACR; n = 3,621) or the hepatic steatosis index (HSI; n = 775). Consequently, 17,229 participants were included in the final analysis. Among them, 3,795 individuals met the diagnostic criteria for metabolic dysfunction–associated steatotic liver disease (MASLD). The detailed recruitment and exclusion flowchart is presented in **Figure 1**.

### 2.2 Data collection and measurements

Sociodemographic characteristics, medical history, and lifestyle factors, including smoking status and alcohol consumption, were collected via standardized self-reported questionnaires. Anthropometric parameters were assessed according to established protocols, and body mass index (BMI) was calculated as body weight (kg) divided by height squared (m²). Blood pressure was measured using a standardized sphygmomanometer after participants had rested in a seated position. Blood samples were obtained after an overnight fast (minimum 8 hours) and analyzed at a central certified laboratory. Spot urine samples were used to determine urinary albumin and creatinine concentrations, from which the urine albumin-to-creatinine ratio (UACR) was calculated.

### 2.3 Definition of MASLD

MASLD was defined in accordance with the recent multi-society Delphi consensus statement on steatotic liver disease nomenclature, which requires the presence of hepatic steatosis together with at least one cardiometabolic risk factor (1). Hepatic steatosis was identified using the hepatic steatosis index (HSI), a non-invasive surrogate marker based on the ALT/AST ratio, body mass index, sex, and diabetes status. HSI has been validated in a Korean adult population, demonstrating good diagnostic performance (AUROC = 0.812; specificity = 92.4% at the ≥36.0 cutoff) (14). To ensure high diagnostic specificity for epidemiologic classification, participants with HSI ≥36.0 were defined as having hepatic steatosis, whereas those with intermediate (30.0≤ HSI <36.0) or low values (HSI <30.0) were categorized as non-steatotic. The cardiometabolic risk factors were defined as follows: obesity (BMI ≥23 kg/m² or waist circumference ≥90/80 cm for men/women), impaired glucose metabolism (HbA1c ≥5.7%, fasting plasma glucose ≥100 mg/dL, a prior diagnosis of type 2 diabetes mellitus, or the use of antidiabetic medications), hypertriglyceridemia (serum triglycerides ≥150 mg/dL), low HDL cholesterol (<40/50 mg/dL for men/women), and elevated blood pressure (systolic/diastolic BP ≥130/85 mmHg or the use of antihypertensive medications). To ensure the specificity of the MASLD diagnosis, only those with no or mild alcohol consumption (as defined in the exclusion criteria) were included in the MASLD cohort.

### 2.4 Assessment of liver fibrosis and CKD

The severity of liver fibrosis was assessed using the Fibrosis-4 (FIB-4) index, calculated from age, aspartate aminotransferase (AST), alanine aminotransferase (ALT), and platelet count (15). Based on established cut-off values, participants were initially categorized into three risk groups: low (<1.3), intermediate (1.3–2.67), or high (>2.67) risk of advanced fibrosis. To address the right-skewed distribution of FIB-4 values and ensure statistical robustness, log-transformed FIB-4 was also employed in continuous variable analyses.

CKD was defined as an estimated glomerular filtration rate (eGFR) <60 mL/min/1.73 m² or a UACR ≥30 mg/g creatinine (16). The eGFR was calculated using the Chronic Kidney Disease Epidemiology Collaboration (CKD-EPI) equation (17). To further characterize the nature of renal impairment, we additionally analyzed CKD components separately as reduced eGFR (eGFR <60 mL/min/1.73 m²) and albuminuria (UACR ≥30 mg/g), representing functional decline and structural kidney injury, respectively.

### 2.5 Statistical analysis

All analyses were conducted using SAS 9.4 (SAS Institute Inc., Cary, NC, USA). To ensure national representativeness, the complex sampling design of KNHANES was strictly accounted for by incorporating stratification, clustering, and survey weights in all statistical procedures. Continuous variables were presented as mean ± standard errors, while categorical variables were expressed as frequencies and weighted percentages. Differences in baseline characteristics according to the MASLD status were evaluated using survey-weighted t-tests for continuous data and the Rao–Scott chi-square test for categorical data. The association between MASLD and prevalent CKD was examined using survey-weighted logistic regression models to calculate odds ratios (ORs) and 95% confidence intervals (CIs). The associations were evaluated for the composite CKD outcome as well as separately for reduced eGFR and albuminuria using the same modeling approach. Multivariate models were constructed by adjusting for potential confounders, including age, sex, income level, education years, alcohol consumption, smoking status, and the risk of advanced liver fibrosis. To verify the robustness of our primary results, sensitivity analyses were performed by redefining hepatic steatosis using the Korea Nonalcoholic Fatty Liver Disease (K-NAFLD) score, an alternative steatosis index incorporating sex, waist circumference, systolic blood pressure, fasting serum glucose, triglycerides, and alanine aminotransferase (18). We also repeated the fibrosis analyses using the AST-to-Platelet Ratio Index (APRI), an alternative non-invasive fibrosis marker based on AST and platelet count that does not incorporate age (19). Similar to the primary FIB-4 analysis, log-transformed APRI was analyzed as a continuous variable after adjustment for age and the same potential confounders. Furthermore, potential effect modification was assessed through subgroup analyses and formal tests for interaction across age, sex, and lifestyle factors. Within the MASLD group, the additive interaction between hypertension and diabetes was evaluated using the relative excess risk due to interaction (RERI) and the attributable proportion (AP). In addition, the correlation between log-transformed FIB-4 and eGFR was evaluated using survey-weighted linear regression. Given the confounding influence of age on both fibrosis indices and renal function, age-stratified logistic regression was further employed to assess the relationship between log FIB-4 and prevalent CKD. A two-sided P value <0.05 was considered statistically significant.

## 3 Results

### 3.1 Baseline characteristics

The baseline clinical and socioeconomic characteristics of the study population, stratified by the presence of MASLD, are summarized in **Table 1**. The total cohort had a mean age of 51.4 ± 16.3 years, with no significant age-related disparity observed between the MASLD and non-MASLD groups. Compared to the non-MASLD group, individuals with MASLD were more likely to be male and exhibited a distinct socioeconomic profile, characterized by significantly lower income levels and fewer years of formal education. Notably, the MASLD group displayed a markedly more adverse cardiometabolic profile than the non-MASLD group. This included a substantially higher prevalence of key metabolic comorbidities, such as hypertension, diabetes mellitus, and dyslipidemia. Regarding renal function, the MASLD group was characterized by a significantly lower mean estimated glomerular filtration rate (eGFR) and a higher burden of metabolic derangements compared to their non-MASLD counterparts. These findings underscore the clustering of cardiometabolic risk factors within the MASLD phenotype.

**Table 1:** Baseline clinical characteristics in non-MASLD and MASLD.

Characteristics	Total population (N = 17,229)	Non-MASLD group (n=13,434)	MASLD group (n=3,795)	*P* value
Age (years)	51.4 (16.3)	51.4 (16.7)	51.3 (15.0)	0.764
Sex, n (%)				< 0.001
Male	6,673 (38.7)	5,100 (38.0)	1,573 (41.4)	
Female	10,556 (61.3)	8,334 (62.0)	2,222 (58.6)	
Income level, n (%)				< 0.001
< 25%	4,080 (23.9)	3,113 (23.4)	967 (25.7)	
25 – 49%	4,296 (25.2)	3,298 (24.8)	998 (26.5)	
50 – 74%	4,312 (25.3)	3,374 (25.3)	938 (24.9)	
≥ 75%	4,384 (25.7)	3,525 (26.5)	859 (22.8)	
Education years, n (%)				< 0.001
≤ 6	4,232 (26.0)	3,218 (25.3)	1,014 (28.2)	
7 – 9	1,752 (10.7)	1,305 (10.3)	447 (12.4)	
10 – 12	5,354 (32.8)	4,212 (33.1)	1,142 (31.8)	
> 12	4,968 (30.5)	3,975 (31.3)	993 (27.6)	
Medical history, n (%)				
Hypertension	4,927 (30.2)	3,352 (26.3)	1,575 (43.8)	< 0.001
Diabetes	2,348 (13.6)	1,182 (8.8)	1,166 (30.7)	< 0.001
Dyslipidemia	6,019 (35.6)	3,969 (30.0)	2,050 (55.4)	< 0.001
Viral hepatitis B	619 (3.6)	484 (3.6)	135 (3.6)	0.894
Body mass index (kg/m^2^)	23.7 (3.4)	22.6 (2.5)	27.8 (3.0)	< 0.001
Waist circumference (cm)	80.8 (9.8)	78.0 (8.2)	90.8 (8.0)	< 0.001
Systolic BP (mmHg)	118.3 (16.9)	117 (17.0)	123 (16.0)	< 0.001
Diastolic BP (mmHg)	74.8 (10.1)	73.8 (9.9)	78.3 (10.1)	< 0.001
HbA1c (%)	5.8 (0.8)	5.7 (0.7)	6.2 (1.1)	< 0.001
Fasting blood glucose (mg/dL)	98.7 (22.2)	95.6 (17.5)	109.5 (31.7)	< 0.001
Total cholesterol (mg/dL)	189.5 (36.0)	187.5 (34.9)	196.7 (38.9)	< 0.001
Triglyceride (mg/dL)	127.7 (91.1)	116.0 (80.7)	169.2 (111.7)	< 0.001
HDL cholesterol (mg/dL)	50.2 (12.0)	51.5 (12.1)	45.9 (10.5)	< 0.001
LDL cholesterol (mg/dL)	114.4 (31.8)	113.3 (30.8)	118.5 (34.6)	< 0.001
AST (U/L)	21.8 (11.0)	20.7 (9.4)	25.5 (14.5)	< 0.001
ALT (U/L)	20.8 (17.4)	17.0 (9.6)	34.2 (28.6)	< 0.001
Glomerular filtration rate (mL/min/1.73 m^2^)	96.5 (16.9)	96.7 (17.0)	95.8 (16.5)	0.003
Hepatic steatosis index	32.5 (5.1)	30.5 (3.2)	39.8 (3.7)	< 0.001
FIB-4 high risk, n (%)	413 (2.4%)	372 (2.8%)	41 (1.1%)	< 0.001
Alcohol history, n (%)				0.001
Non drinker	9,071 (55.3)	6,981 (54.6)	2,090 (57.7)	
Social drinker	7,346 (44.7)	5,813 (45.4)	1,533 (42.3)	
Smoking history, n (%)				< 0.001
Non smoker	11,100 (66.4)	8,738 (67.0)	2,362 (64.1)	
Ever smoker	5,626 (33.6)	4,306 (33.0)	1,320 (35.9)	

Values are expressed as mean (standard errors) or number (%).

MASLD, metabolic dysfunction-associated steatotic liver disease; BP, blood pressure; DBP, diastolic blood pressure; HbA1c, hemoglobin A1c; HDL, high-density lipoprotein; LDL, low-density lipoprotein; AST, aspartate aminotransferase; ALT, alanine aminotransferase; HSI, hepatic steatosis index; FIB-4, fibrosis-4 index.

### 3.2 Association of MASLD with prevalent CKD

To evaluate the association of MASLD with renal health, the study population was categorized into three groups based on the presence of steatotic liver disease (SLD) and cardiometabolic risk factors (CMRF) (**Table 2**). Compared with the reference group (no SLD without CMRF), MASLD was associated with prevalent CKD after multivariable adjustment (OR, 2.47; 95% CI, 1.76–3.47; p < 0.001). Notably, individuals with CMRF but without SLD also had significantly higher odds of prevalent CKD (OR, 1.41; 95% CI, 1.03–1.95; p = 0.034), although the strength of the association was lower than that observed in the MASLD group.

**Table 2:** Association of MASLD and cardiometabolic risk factors with prevalent CKD.

Study group/CMRF	Composite CKD (reduced eGFR or albuminuria)			Reduced eGFR (<60 mL/min/1.73 m²)			Albuminuria (UACR ≥ 30 mg/g)		
	Events, n (%)	Odds ratio (95% CI)	*P* value	Events, n (%)	Odds ratio (95% CI)	*P* value	Events, n (%)	Odds ratio (95% CI)	*P* value
Total population									
No SLD without CMRF	73 (2.5)	Reference		6 (0.1)	Reference		70 (2.5)	Reference	
No SLD with any CMRF	1116 (8.3)	1.41 (1.03-1.95)	0.034	376 (2.4)	4.62 (1.76-12.15)	0.002	883 (6.9)	1.41 (1.02-1.95)	0.038
MASLD	523 (11.1)	2.47 (1.76-3.47)	<0.001	115 (2.1)	6.87 (2.55-18.54)	<0.001	454 (10.1)	2.52 (1.79-3.55)	<0.001
MASLD									
Overweight or obesity	517 (11.2)	1.72 (0.67-4.45)	0.262	113 (2.1)	1.31 (0.25-6.78)	0.747	449 (10.2)	1.90 (0.69-5.21)	0.213
Dysglycemia or type 2 diabetes	475 (14.2)	1.85 (1.23-2.78)	0.003	114 (3.0)	24.96 (3.36-185.63)	0.002	407 (12.7)	1.97 (1.32-2.96)	0.001
Plasma triglyceride	274 (12.8)	1.26 (0.97-1.64)	0.086	59 (2.1)	1.53 (0.89-2.63)	0.128	240 (11.9)	1.45 (1.12-1.87)	0.005
HDL-cholesterol	307 (12.4)	0.97 (0.74-1.27)	0.825	74 (2.7)	1.10 (0.71-1.69)	0.675	265 (11.2)	1.10 (0.85-1.42)	0.482
Elevated blood pressure	426 (17.7)	2.77 (2.01-3.81)	<0.001	101 (3.7)	4.22 (1.83-9.69)	<0.001	366 (15.9)	2.75 (2.02-3.75)	<0.001

The model was adjusted for age, sex, income level, education years, alcohol/smoking status, and risk of advanced liver fibrosis.

Events are presented as unweighted counts, whereas percentages are weighted proportions accounting for the complex survey design.

CKD, chronic kidney disease; eGFR, estimated glomerular filtration rate; UACR, urine albumin-to-creatinine ratio; SLD, steatotic liver disease; CMRF, cardiometabolic risk factor; MASLD, metabolic dysfunction-associated steatotic liver disease; CI, confidence interval.

When CKD components were analyzed separately, MASLD remained significantly associated with both reduced eGFR (OR, 6.87; 95% CI, 2.55–18.54; p < 0.001) and albuminuria (OR, 2.52; 95% CI, 1.79–3.55; p < 0.001), showing consistent patterns across outcomes.

**Figure 2:**
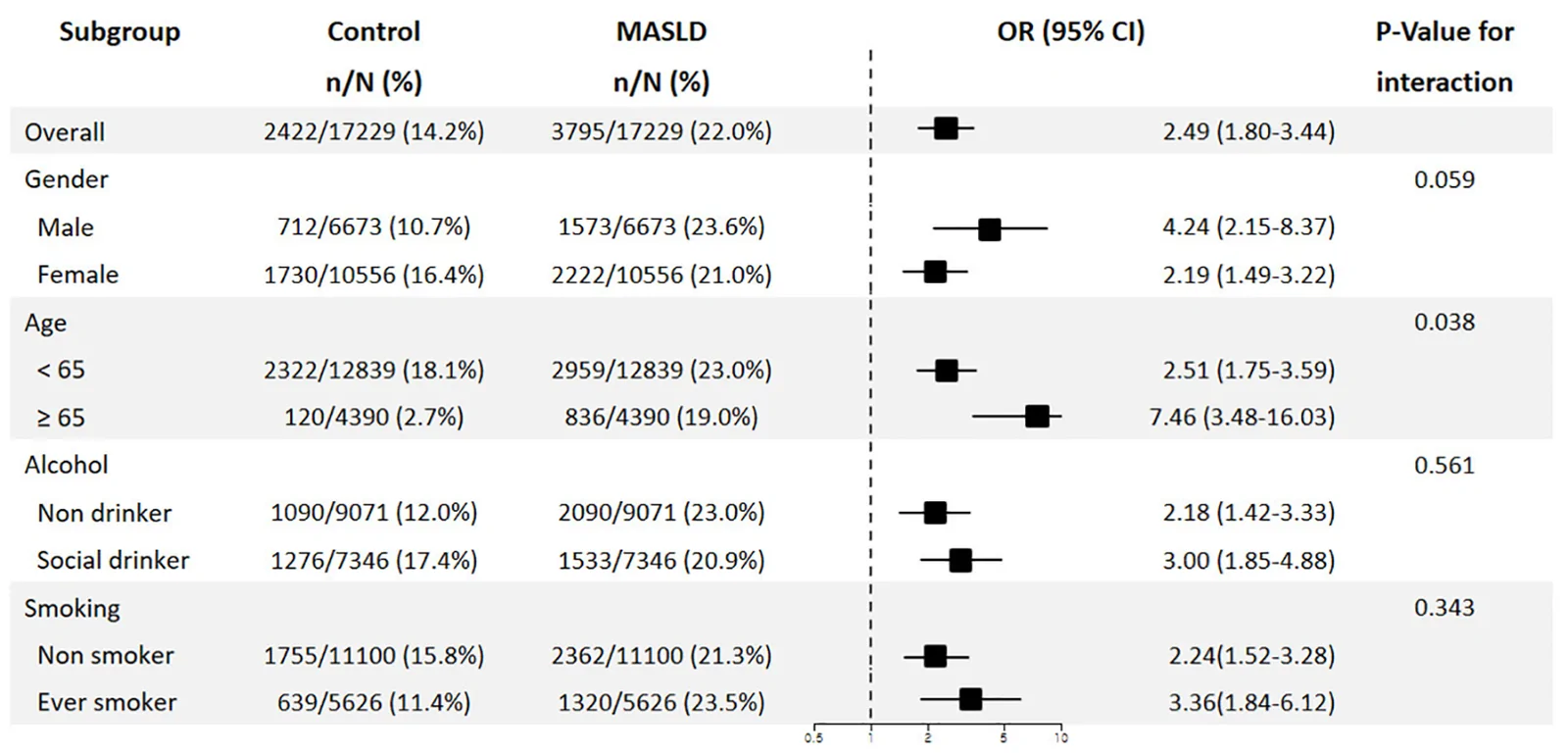
Subgroup analyses of the association between MASLD and prevalent CKD. Forest plot showing adjusted odds ratios for prevalent CKD according to subgroups defined by age, sex, alcohol consumption, and smoking status. A significant interaction was observed with age (P for interaction = 0.038). Models were adjusted for age, sex, income level, education years, alcohol/smoking status, and risk of advanced liver fibrosis. MASLD, metabolic dysfunction-associated steatotic liver disease; OR, odds ratio; CI, confidence interval.

Subgroup analyses stratified by age, sex, alcohol consumption, and smoking status are presented in **Figure 2**. A significant interaction was observed between MASLD and age (P for interaction = 0.038). The association between MASLD and CKD was substantially stronger in elderly participants (age ≥65 years; OR, 7.46) compared to those under 65 (OR, 2.51). To ensure the robustness of our findings, sensitivity and weighted analyses were performed. Sensitivity analyses utilizing the K-NAFLD score as an alternative steatosis definition yielded consistent and significant estimates (OR, 3.88; 95% CI, 2.72–5.53; p < 0.001) (**Supplementary Table 1**). Furthermore, propensity score–based inverse probability of treatment weighting (IPTW) produced similar results, with a strengthened association in the weighted multivariate model (OR, 5.12; 95% CI, 3.68–7.14; p < 0.001) (**Supplementary Table 2**). Collectively, these diverse analytic approaches support the consistency and reliability of the primary findings.

### 3.3 Metabolic components and fibrosis severity in MASLD

We further investigated the MASLD cohort to identify the associations between individual cardiometabolic components and prevalent CKD (**Table 2**). In the fully adjusted model, elevated blood pressure and dysglycemia or type 2 diabetes were consistently associated with all three renal outcomes – composite CKD, reduced eGFR, and albuminuria – whereas other metabolic components were not. To explore potential synergistic effects, additive interaction analyses were performed. While a positive direction was observed in the additive interaction between hypertension and diabetes, the results did not reach statistical significance, with a RERI of 0.60 (95% CI, -1.25–2.36; p = 0.454) and an AP of 0.10 (95% CI, -0.19–0.38; p = 0.423). These results suggest that while a positive numerical direction for the interaction was observed, the synergistic effect did not reach statistical significance in the current cohort.

**Figure 3:**
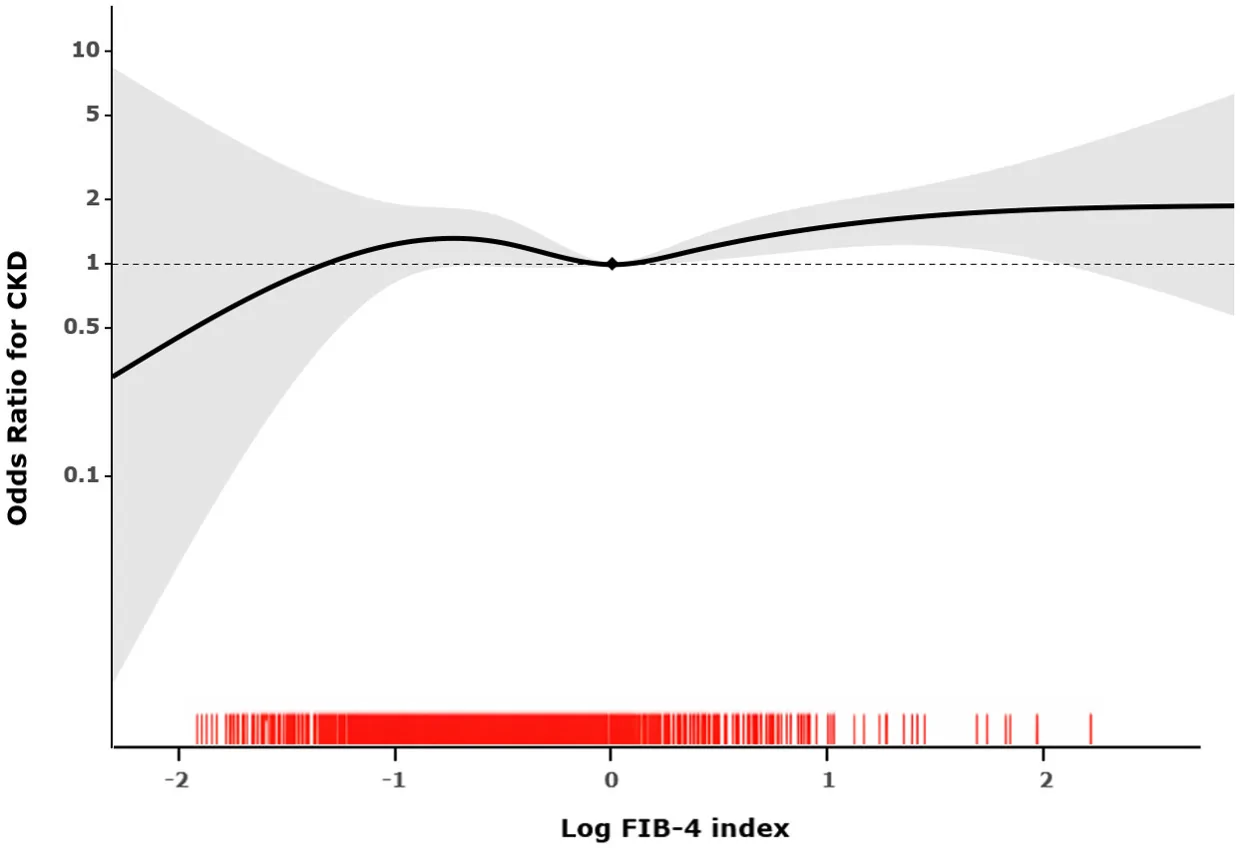
Graded association of liver fibrosis severity with prevalent CKD in MASLD. Restricted cubic spline showing the association between log-transformed Fibrosis-4 (FIB-4) index and the odds of prevalent CKD. The solid line represents adjusted odds ratios, and the shaded area indicates 95% confidence intervals. The reference value was set at log FIB-4 = 0. Models were adjusted for age, sex, income level, education years, and alcohol/smoking status. CKD, chronic kidney disease; FIB-4, fibrosis-4 index.

Next, we evaluated the relationship between liver fibrosis severity and renal function using log-transformed FIB-4 index. Scatter plots demonstrated an inverse correlation between log FIB-4 and eGFR (**Supplementary Figure 1**). In addition, restricted cubic spline analysis showed a graded association, where increasing fibrosis severity corresponded to a higher prevalence of CKD (**Figure 3**). Given that age is an inherent component of the FIB-4 index, we conducted age-stratified analyses to account for potential confounding (**Table 3**). In the middle-aged group (40–64 years), higher log FIB-4 levels were notably associated with prevalent CKD (OR, 1.88; 95% CI, 1.18–2.98; p = 0.007). While similar trends were observed in the younger (<40 years) and older (≥65 years) groups, these findings did not reach statistical significance (p = 0.079 and p = 0.082, respectively). These results suggest that the association between liver fibrosis and renal function may vary across age groups, appearing most prominent during the transitional phase of middle adulthood. Sensitivity analyses utilizing log-transformed APRI as an alternative fibrosis marker yielded consistent results, with higher log-transformed APRI remaining significantly associated with prevalent CKD in the overall MASLD population (adjusted OR, 1.38; 95% CI, 1.02–1.85; p = 0.035) (**Supplementary Table 3**).

**Table 3:** Age-stratified association between log-transformed FIB-4 index and prevalent CKD in MASLD.

Age group	OR per 1-unit increase in log FIB-4	95% CI	*P* value
< 40	2.34	0.91 – 6.03	0.079
40 – 64	1.88	1.18 – 2.98	0.007
≥ 65	1.60	0.94 – 2.70	0.082

The model was adjusted for sex, income level, education years, alcohol/smoking status.

OR, odds ratio; FIB-4, fibrosis-4 index; CI, confidence interval.

## 4 Discussion

In this nationwide population-based study utilizing data from the KNHANES 2011–2014, we found that metabolic dysfunction-associated steatotic liver disease (MASLD) is associated with a higher prevalence of chronic kidney disease (CKD). Our analysis highlights the phenotypic heterogeneity within the MASLD framework, whereby the association with CKD varies according to the burden of cardiometabolic risk factors and the severity of liver fibrosis. Within the MASLD cohort, hypertension and dysglycemia/type 2 diabetes were consistently associated with CKD across multiple outcome definitions, whereas other metabolic components showed less consistent associations. Although a positive direction of additive interaction between hypertension and diabetes was observed, the interaction did not reach statistical significance. In addition, we observed a graded association between liver fibrosis severity and CKD prevalence. This association appeared to vary across age groups, with a more evident association observed among middle-aged adults.

The link between steatotic liver disease and renal dysfunction has been consistently reported across evolving disease definitions. Previous research under the NAFLD (non-alcoholic fatty liver disease) framework established that hepatic steatosis was associated with both prevalent and incident CKD, independent of traditional cardiometabolic risk factors (20). Subsequent meta-analyses further corroborated these findings, reporting that NAFLD confers approximately 1.4 to 1.6-fold increased risk of CKD, with the magnitude of risk escalating in tandem with the advancement of liver disease (8, 21). Conceptual frameworks by Targher, Byrne, and colleagues have positioned NAFLD as a systemic metabolic disorder, suggesting that its renal implications extend beyond shared comorbidities (22, 23).

With the 2020 introduction of the MAFLD (metabolic dysfunction-associated fatty liver disease) definition, which shifted the emphasis toward positive metabolic criteria, several cohorts reported a similar or even superior capacity for renal risk stratification compared to the NAFLD definition (24, 25). The most recent MASLD nomenclature further refines this paradigm by integrating a more precise classification of metabolic risk and alcohol consumption (1). Our study contributes to this evolving field by validating the MASLD–CKD association in a large-scale, representative general population.

Beyond the mere presence of steatosis, the severity of hepatic involvement – specifically the degree of fibrosis – serves as a critical determinant of systemic outcomes. Accumulating evidence identifies liver fibrosis as the most robust predictor of long-term prognosis in patients with steatotic liver disease. A landmark meta-analysis underscored that the stage of fibrosis, rather than the grade of steatosis, is the key factor in adverse clinical events (21). Consistent with this, longitudinal cohort studies have demonstrated that advanced fibrosis independently accelerates the development of CKD (26, 27). These associations remain consistent under the MAFLD framework, where elevated non-invasive fibrosis scores were significantly correlated with renal impairment (24). Collectively, these findings reinforce the role of liver fibrosis as a pivotal prognostic axis linking hepatic pathology to adverse renal outcomes.

Building upon these observations, the present study extends the current understanding by elucidating the heterogenous patterns of CKD risk within the MASLD spectrum. While prior investigations often characterized steatotic liver disease as a binary exposure, our results reveal that individual metabolic components do not contribute equally to the prevalence of CKD. In particular, elevated blood pressure and dysglycemia or type 2 diabetes were consistently associated with CKD across multiple outcome definitions. Although point estimates suggested a positive direction for additive interaction between these components, the interaction did not reach statistical significance. This finding should be interpreted with caution, as the limited number of events in this population-based cohort may have reduced the statistical power to detect interaction. Our results are in line with contemporary European recommendations advocating stepwise case finding and fibrosis risk stratification in individuals with MASLD, particularly those with type 2 diabetes, obesity, or multiple cardiometabolic risk factors (28, 29). These observations further highlight the clinical relevance of integrating metabolic risk profiling with fibrosis assessment in the evaluation of patients with MASLD.

From a pathophysiological perspective, hypertension and diabetes are known to affect renal function through overlapping but distinct mechanisms. Hypertension contributes to glomerular hypertension and endothelial dysfunction, whereas diabetes is associated with advanced glycation end-products, oxidative stress, and podocyte injury. Within the context of systemic metabolic dysregulation in MASLD, these pathways may jointly contribute to renal impairment. This observation aligns with the broader paradigm that metabolic syndrome acts as a multisystemic factor associated with CKD progression, supporting the concept of MASLD as a hepatic manifestation of systemic metabolic dysfunction (30).

Age-stratified analyses provided further insight into the heterogeneity in the MASLD–CKD association across the life course. Age is a potent determinant of both renal function and non-invasive fibrosis indices, such as the FIB-4 index. Consequently, in unstratified models, the observed link between liver fibrosis and CKD can be attenuated by age-related confounding.

We found that the association between fibrosis severity and CKD was more evident among middle-aged adults (40–64 years). This pattern is consistent with epidemiological observations showing an escalation in CKD burden after midlife, where the convergence of metabolic syndrome and aging-related processes relates to nephron loss (31–33). In contrast, among individuals younger than 40 years, higher baseline eGFR and preserved renal reserve may limit the detection of early renal dysfunction, resulting in non-significant associations. Among older adults (≥65 years), the attenuation of statistical significance may reflect the influence of age-related decline in kidney function and a higher burden of comorbidities. Nevertheless, the comparable magnitude of effect estimates in the older group suggests that liver fibrosis may remain a relevant marker of renal impairment in older adults.

Several complex biological pathways likely underpin the observed liver–kidney axis in MASLD. Compared with individuals who had cardiometabolic risk factors without steatotic liver disease, participants with MASLD exhibited a substantially higher prevalence of CKD, suggesting that hepatic steatosis may reflect a more severe systemic metabolic phenotype. This condition is characterized by a milieu of chronic low-grade inflammation, insulin resistance, and pathophysiological activation of the renin–angiotensin–aldosterone system, all of which are known to contribute to endothelial dysfunction and altered adipokine profiles (22, 23, 34). The emerging concept of “fatty kidney” -characterized by ectopic lipid accumulation within renal tissue-further highlights the shared lipotoxic mechanisms that link hepatic and renal injury (35).

Experimental and clinical evidence suggests that systemic metabolic inflammation and oxidative stress do not act in isolation; rather, they simultaneously promote hepatic fibrogenesis and renal tubulointerstitial fibrosis (34, 36). At the molecular level, dysregulated long non-coding RNAs may modulate hepatic stellate cell activation, inflammatory signaling, oxidative stress, and lipid metabolism, thereby contributing to hepatic fibrosis (37). Furthermore, recent studies have identified gut microbiota dysbiosis and systemic release of circulating pro-fibrotic mediators as key contributors to these bidirectional hepatorenal interactions (38, 39). When integrated, these diverse pathways underscore the intrinsically interconnected nature of hepatic and renal injury, positioning MASLD as a systemic condition closely linked to CKD rather than a localized liver pathology.

The primary strength of this study lies in its use of the KNHANES database with complex sampling weights, which provides nationally representative estimates of the MASLD–CKD relationship that are highly applicable to general clinical practice. Furthermore, this study adds to existing evidence by evaluating additive interaction between metabolic components and examining the association of fibrosis severity across different age strata.

Nevertheless, several limitations warrant consideration. First, the cross-sectional design, based on single-point measurements, precludes the determination of temporal relationships between MASLD and CKD. Consequently, causality cannot be definitively inferred, and the possibility of reverse causation remains. Potential sources of bias, including residual confounding inherent to the cross-sectional design, were considered. Chronic kidney disease itself can exacerbate systemic inflammation, oxidative stress, and insulin resistance, all of which are key factors in the development and progression of hepatic steatosis and fibrosis. Therefore, the relationship between MASLD and CKD is likely to be bidirectional. Second, hepatic steatosis was identified using the hepatic steatosis index (HSI), a validated non-invasive surrogate marker, rather than imaging modalities or liver biopsy. Although HSI has demonstrated good diagnostic performance among Korean adults, it remains an indirect measure of hepatic steatosis. Therefore, participants with mild hepatic steatosis, particularly those with intermediate HSI values, may have been misclassified. To address this limitation, sensitivity analyses using the Korean-specific K-NAFLD score yielded consistent findings, supporting the robustness of our results despite the use of surrogate steatosis indices. In addition, because both the HSI and the K-NAFLD score incorporate cardiometabolic variables that are themselves associated with CKD, the independent contribution of hepatic steatosis itself cannot be completely separated from the underlying metabolic abnormalities. Liver fibrosis was assessed using the FIB-4 index, which incorporates age into its calculation. Although age-stratified analyses were performed, residual mathematical coupling with age cannot be completely excluded. Moreover, clinically significant liver fibrosis may be present despite persistently normal ALT levels in patients with MASLD, underscoring the need for cautious interpretation of ALT-containing non-invasive fibrosis markers (40). To address this concern, we performed an additional sensitivity analysis using the AST-to-Platelet Ratio Index (APRI), an age-independent fibrosis marker. The association between log-transformed APRI and prevalent CKD remained significant after adjustment for age and other potential confounders, providing additional support for the primary analysis. Third, the reduced eGFR analyses were based on a relatively small number of outcome events that were highly concentrated in several metabolic exposure groups. Consequently, some effect estimates, particularly for dysglycemia or type 2 diabetes, were numerically large and accompanied by wide confidence intervals. These estimates should therefore be interpreted with appropriate caution and warrant confirmation in larger prospective studies. Fourth, alcohol consumption was assessed via self-reported surveys, which are inherently susceptible to recall or social desirability bias. Since the MASLD classification relies on specific alcohol intake thresholds, inaccurate reporting could lead to misclassification of MASLD and other steatotic liver disease subtypes, such as MetALD. Additionally, information on autoimmune hepatitis and drug-induced liver injury was unavailable in the KNHANES database, and their potential influence on HSI-based classification could not be completely excluded. Finally, our study population consisted exclusively of Korean adults, reflecting the specific metabolic and genetic profiles of an East Asian population. Given that adiposity patterns, insulin resistance, alcohol metabolism, and CKD susceptibility vary significantly across ethnic groups, caution is warranted when generalizing these findings to other populations. Prospective longitudinal studies and external validation in diverse global cohorts are necessary to clarify the generalizability and causal implications of our findings.

## 5 Conclusions

This nationwide population-based study demonstrates that MASLD is associated with an increased prevalence of CKD among Korean adults. This association varies according to metabolic profiles, particularly the co-occurrence of hypertension and dysglycemia, and according to the severity of liver fibrosis. The association between liver fibrosis and CKD is most evident in middle-aged individuals. These results underscore the importance of risk stratification for identifying individuals with prevalent CKD among individuals with MASLD.

## Data Availability

The raw data supporting the conclusions of this article will be made available by the authors, without undue reservation.

## Supplementary material

Supplementary Table 1 Sensitivity analysis using the K-NAFLD score to define hepatic steatosis.

Supplementary Table 2 Propensity score-weighted analysis (IPTW).

Supplementary Table 3 Sensitivity analysis using the APRI to assess liver fibrosis.

Supplementary Figure 1 Relationship between liver fibrosis severity and renal function in MASLD.
