# Supplementary material for: Association of MASLD with chronic kidney disease according to metabolic profiles and fibrosis severity: a KNHANES analysis
Source: Front Endocrinol (Lausanne). 2026 Sep 1;17:1923883. doi: 10.3389/fendo.2026.1923883 (PMC13574625; doi:10.3389/fendo.2026.1923883)
Supplement: Supplementary file 1 [file SupplementaryFile1.docx]

**Supplementary Materials**

Table S1. Sensitivity analysis using the K-NAFLD score to define hepatic steatosis...........p. 2

Table S2. Propensity score-weighted analysis (IPTW)..........................................................p. 3

Table S3. Sensitivity analysis using the APRI to assess liver fibrosis...................................p. 4

Figure S1. Relationship between liver fibrosis severity and renal function in MASLD........p. 5

**Table S1. Sensitivity analysis using the K-NAFLD score to define hepatic steatosis**

|  | **Univariate analysis** | | | **Multivariate analysis^*^** | |
| --- | --- | --- | --- | --- | --- |
|  | **Odds ratio (95% CI)** | | ***P* value** | **Odds ratio (95% CI)** | ***P* value** |
| **Total population** |  |  | |  |  |
| No SLD without CMRF | Reference |  | | Reference |  |
| No SLD with any CMRF | 3.27 (2.43-4.40) | <0.001 | | 1.41 (1.03-1.94) | 0.034 |
| MASLD | 8.51 (6.16-11.74) | <0.001 | | 3.88 (2.72-5.53) | <0.001 |
| **MASLD** |  |  | |  |  |
| Overweight or obesity | 0.77 (0.45-1.32) | 0.340 | | 1.09 (0.62-1.94) | 0.758 |
| Dysglycemia or type 2 diabetes | 2.84 (1.79-4.49) | <0.001 | | 1.83 (1.13-2.97) | 0.014 |
| Plasma triglyceride | 1.26 (0.90-1.77) | 0.174 | | 1.27 (0.96-1.68) | 0.097 |
| HDL-cholesterol | 1.14 (0.85-1.53) | 0.378 | | 0.91 (0.64-1.28) | 0.576 |
| Elevated blood pressure | 3.33 (2.34-4.74) | <0.001 | | 2.49 (1.67-3.71) | <0.001 |

^*^ The model was adjusted for age, sex, income level, education years, alcohol/smoking status, and risk of advanced liver fibrosis.

Abbreviations: SLD = steatotic liver disease; CMRF = cardiometabolic risk factor; MASLD = metabolic dysfunction-associated steatotic liver disease; CI = confidence interval.

**Table S2. Propensity score-weighted analysis (IPTW)**

|  | **Univariate analysis** | | | **Multivariate analysis^*^** | |
| --- | --- | --- | --- | --- | --- |
|  | **Odds ratio (95% CI)** | | ***P* value** | **Odds ratio (95% CI)** | ***P* value** |
| **Total population** |  |  | |  |  |
| No SLD without CMRF | Reference |  | | Reference |  |
| No SLD with any CMRF | 3.48 (2.56-4.73) | <0.001 | | 3.53 (2.58-4.83) | <0.001 |
| MASLD | 4.73 (3.42-6.54) | <0.001 | | 5.12 (3.68-7.14) | <0.001 |

^*^ The model was adjusted for age, sex, income level, education years, alcohol/smoking status, and risk of advanced liver fibrosis.

Abbreviations: SLD = steatotic liver disease; CMRF = cardiometabolic risk factor; MASLD = metabolic dysfunction-associated steatotic liver disease; CI = confidence interval.

**Table S3. Sensitivity analysis using the APRI to assess liver fibrosis**

| **Age group** | **OR per 1-unit increase in log APRI** | **95% CI** | ***P* value** |
| --- | --- | --- | --- |
| Overall | 1.38 | 1.02 – 1.85 | 0.035 |
| < 40 | 1.27 | 0.61 – 2.65 | 0.524 |
| 40 – 64 | 1.57 | 1.04 – 2.38 | 0.034 |
| ≥ 65 | 1.39 | 0.93 – 2.08 | 0.110 |

^*^ The model was adjusted for age, sex, income level, education years, alcohol/smoking status.

Abbreviations: OR = odds ratio; APRI = AST-to-Platelet Ratio Index; CI = confidence interval.

**Fig. S1. Relationship between liver fibrosis severity and renal function in MASLD**

Scatter plot showing the correlation between log-transformed Fibrosis-4 (FIB-4) index and estimated glomerular filtration rate (eGFR).

Abbreviations: eGFR = estimated glomerular filtration rate; FIB-4 = fibrosis-4 index.
